# The analysis of factors affecting municipal employees’ willingness to report to work during an influenza pandemic by means of the extended parallel process model (EPPM)

**DOI:** 10.1186/s12889-015-2663-8

**Published:** 2016-01-12

**Authors:** Carolin von Gottberg, Silvia Krumm, Franz Porzsolt, Reinhold Kilian

**Affiliations:** 1 Department of Psychiatry and Psychotherapy II, Section of Health Economics and Mental Health Services Research, Ulm University, Ludwig-Heilmeyer-Str. 2, D-89315 Günzburg, Germany; 2 Department of General and Visceral Surgery, Working Group of Health Services Research, Ulm University, Albert-Einstein-Allee 23, D-89070 Ulm, Germany

**Keywords:** Influenza pandemic, Municipal employees, Preparedness, Willingness to report to work, Extended parallel process model, Critical infrastructure, Germany

## Abstract

**Background:**

The management of pandemics with highly infectious diseases in modern urban habitats depends largely on the maintenance of public services. Understanding the factors that influence municipal employees’ willingness to come to work during a pandemic is therefore a basic requirement for adequate public health preparedness. In this study the extended parallel process model (EPPM) is applied to investigate how the readiness of municipal employees to report to work during an influenza pandemic (IP) is affected by individual attitudes and environmental conditions.

**Methods:**

1.566 employees of a major German city participated in a cross-sectional online survey. The questions of the survey covered the dimensions of risk perception, role competence, self-efficacy, role importance, sense of duty, and willingness to report to work in the case of an IP. Data were analysed by means of path analyses.

**Results:**

Data suggest that up to 20 % of the public service workers were not willing to come to work during an IP. Willingness to report to work was increased by the perception of a high working role competence, a high assessment of role importance, high self-efficacy expectations, and a high sense of duty. Negative effects on willingness to report to work were identified as the perception of a high risk to become infected at work and the perceived risk to infect family members. The decomposition of direct and indirect effects provided important insights into the interrelationships between model variables.

**Conclusions:**

Measures to increase municipal workers’ willingness to report to work in case of an infectious pandemic should include communication strategies to inform employees clearly about their particular tasks during such critical events and training exercises to increase their confidence in their competences and skills to fulfil these tasks.

## Background

Current research on pandemic preparedness is mainly focused on health care services. However, maintaining adequate functioning of health care in modern urban settings depends largely on the functioning of the so called critical infrastructure, which includes a broad spectrum of municipal services ranging from water and power supply, public safety, public transport, traffic surveillance, emergency services, garbage removal, sewage disposal, and so on [1–4]. The recent outbreak of the Ebola virus disease in West Africa demonstrates drastically the fatal consequences a collapse of the critical infrastructure in the case of an aggressive disease pandemic has [5–7].

As it has been extensively discussed for health care services [8, 9], keeping up non-medical municipal services during the pandemic of a highly contagious disease is jeopardized by infections of staff members but also by the decision of employees to stay away from work without being ill. The decision not to report to work might be motivated by the intention to avoid being infected or to carry the infection into their families. The understanding of the factors that affect the willingness to report to work during a severe infectious disease pandemic is therefore an essential requirement for municipal emergency planning [4].

Studies in the general population indicated that 20–30 % of the working population wouldn’t leave their homes during an influenza pandemic (IP) and 50 % would ask for time off [10, 11]. In contrast, members of health care services were found to express a much higher willingness to report to duty, even if they were only asked but not required to report by their employers [12–17]. Nevertheless, Damery et al. [18] reported that also in the group of medical professionals there are considerable differences in the willingness and ability to respond to a pandemic threat. Nursing and ancillary staff were less willing and less able to report to work than doctors or first responders [18]. While the general high willingness of health care workers to perform their professional duty during a pandemic is confirmed in many studies, Seale et al. [19] showed that even hospital staff members adjust their willingness to report to work to the severity or to the proximity of the pandemic threat. As indicated by the results of a survey on Australian hospital workers, 83 % (*n* = 899) would still be willing to report to work if there was a patient with influenza symptoms on their ward; if co-workers were infected the number of people showing up at work would decline to 79 % (*n* = 852); only 61 % (*n* = 654) would report to work if a family member would fall ill. Wicker et al. [20] reported that 14 % of the (assumed healthy) staff of a German University Hospital would not be willing to report to work during a pandemic. Also in this study there was a clear difference between the occupational groups: only 6 % of the doctors answered that they would not report to work, whereas 24 % of the administrative employees would stay away. Another 12 % weren’t sure how they would handle the situation [20]. This study also raised another highly relevant topic for pandemic preparedness planning: 36 % of those who would be willing to report to work answered that they would not be able to report because of different barriers (like missing child care or public transportation problems) [20].

A theoretical framework for the analyses of the factors affecting the willingness of medical and other emergency service workers to report to work during a disaster situation is provided by the extended parallel process model (EPPM) [13]. The EPPM was developed for the purpose of improving the effectiveness of health risk communication [21]. Rooted in earlier theories of health protective behaviour, such as the protection motivation theory, the EPPM combines elements of the transactional stress model [22] and self-efficacy theory [23] with the parallel process model of fear and danger control [24]. According to the EPPM the probability of adequate self-protecting behaviour increases, if people appraise the severity of and their own susceptibility to a health risk as high and if they also feel able to perform effective preventive actions. In contrast, perceiving a high health risk but a low ability to perform effective measures for prevention may result in fear control reactions such as the denial of risk or the avoidance of risk information [21].

In their adaptation of the EPPM to the investigation of the willingness of local public health workers to report to work during an IP situation Barnett et al. (2009) proposed that adequate response to disaster situations will depend on the right balance between threat appraisal and self-efficacy expectations [13]. In several studies the authors found that the willingness of hospital staff and other medical emergency service workers to report to duty in the case of an IP was highest in respondents who assessed the dangerousness of the pandemic as high and who were highly confident in their capabilities to perform well in their professional role during the pandemic [12–17, 19, 25–27]. As a consequence of their research results the authors concluded that communication and training strategies to improve the willingness of medical and other emergency service workers to report to work in the event of a disaster situation must include measures to convince workers of the importance of their professional tasks and to increase their confidence in their professional skills [16, 27]. In addition, workers must be supported in managing family responsibilities and occupational health risks and overcoming logistic hurdles such as reaching their designated work place in case of failure of public transport [13, 25, 28].

In this article we apply the EPPM to investigate to what extent the willingness of German municipal service workers to report to work during an IP is affected by the same factors that have been identified for medical service workers. The purpose of this research is to increase the empirical basis for improving the general preparedness for public disaster management.

## Method

### Study sample

We asked the municipal employees (*N* = 5.976) of a German city with about 600.000 inhabitants to participate in an online survey via the municipal intranet. In addition we asked the 670 members of the municipal fire brigade, who had no access to a computer at their workplace, to complete a paper pencil questionnaire.

The survey took place from 14.02.2011 to 25.03.2011.

### Ethics statement

After reviewing the study protocol the ethical committee of the Ulm University waived the need for an ethical approval because the project does neither include patients of medical or psychosocial services nor medical interventions or examinations.

According to German legal provisions the study was approved by the management of the city administration, the local data protection commissioner and by the local staff council (Personalrat). Respondents were informed that their participation in the study is voluntary and that their data is collected and processed completely anonymously. To demonstrate their consent to participate in the study respondents were asked to agree to the statement “I understand the purpose and nature of this study and I am participating voluntarily. I understand that I can withdraw from the study at any time, without any penalty or consequences”.

### Assessment instruments

The questionnaire was developed on the basis of the Johns Hopkins Public Health Infrastructure Response Survey Tool - (JH-PHIRST) [28]. The JH-PHIRST, like most tools and surveys in this field, focuses on medical services workers [8] and had to be adjusted for people working in a city administration. To identify administration specific aspects of the willingness to report to work, four focus groups with 14 municipal employees (two with 7 administrative workers and two with 7 management staff) were conducted on 18.06.2010, 23.06.2010 and 01.07.2010. Participants were informed that the discussion was recorded, that their participation was completely voluntary and that they could leave the discussion at any time. Focus groups were opened with a short description of the project. Then the participants were asked to discuss about the probability and the severity of an influenza pandemic and their professional role in the management of such an event and their preparedness and willingness to fulfil their professional role. Group discussions lasted 90 min on average. All focus groups were audio recorded and fully transcribed. Focus group data were analysed by means of computer aided qualitative content analysis using MAXQDA 11 [29]. Aim of the qualitative content analysis was the identification of personal and situational characteristics which, from the perspective of the participants, affect their willingness to report to work in case of an IP . Therefore all statements referring to perceived barriers against reporting to work or characteristics perceived as increasing the motivation to report to work were coded in rephrased form and thematically sorted in a code tree. Code trees were developed independently by two of the authors (CvG and SK) and compared. Differences between codes were discussed in the research group (CvG, SK, FP, RK) and a final code tree was created based on these discussions. The structure of the final code tree included the following themes: The perceived risk and the severity of an IP; the perceived probability that the employer will ask or require the employees to report to work in case of an IP, the perceived importance of the employees’ role in case of an IP; the employees knowledge of their job demands in case of an IP; the employees perceived preparedness to fulfil their expected job demands in case of an IP; the expectations about the decision making process in case of an IP; the employees assessment of the safety of their families in case of an IP; the employees assessment of their chances to reach their workplaces with regard to the impaired functioning of traffic and public transport in case of an IP; the perceived personal or professional duty to report to work in case of an IP; the assessment of the employers sense of responsibility with regard to the employees’ safety in case of an IP; the assessment of the employers willingness to share information in case of an IP. Results of the content analysis revealed that several questions from the JH-PHIRST questionnaires were also applicable to administration workers. Only questions directly related to the duties of medical staff were completely deleted. On the other hand, focus group participants mentioned the expectation that in case of a pandemic they would be requested to take on duties outside their area of responsibility. Another important aspect with regard to the willingness to report to work was the awareness in many of the participants of a particular responsibility for the safety of the citizens. In addition, the confidence in the care of the superiors for the safety of the employees and in the trustworthiness of the information about the situation provided by the management were raised as important factors for the staff members’ willingness to report to duty. Beyond these factors directly related to the professional role the participants also addressed the fact that the responsibility for the safety of family members could interfere with job duties.

The questionnaire developed on the basis of the JH-PHIRST and the focus groups includes 2 statements to assess the willingness to report to work and 18 statements related to the 5 dimensions of the adjusted EPPM (see Appendix 1). All statements were rated on the following 5 point Likert type answering categories: not true all = 1, rather does not agree = 2, partly agree = 3, mainly true = 4, agrees completely = 5. Mean summary scores were computed for all variables including more than 1 item, otherwise the summary score was coded as missing value.

### Statistical analyses

Due to the low number of items on each scale, the Spearman-Brown formula [30] was used to estimate the reliability of the summary scales for measuring the dimensions of the EPPM.

The propositions of the adjusted EPPM were tested by means of path analyses. Separate path models were computed for the willingness to report to work during an influenza pandemic if required by the agency, and for the willingness to report to work if asked but not required by the agency.

### Exogenous variables

To control for socio-demographic characteristics and for features of the job position the following exogenous variables were included in both path models:

- Endogenous variables (see. Table 1)
  Summary scales of the five dimensions of the adjusted EPPM were used as endogenous variables.
- Parameterization of the path models

**Table 1:** Coding of exogenous variables

Exogenous variables:	Codes/Scales
Sex	female = 0, male = 1
Education	below high school = 0, high school and above = 1
Age	in years
Civil servant status	no = 0, yes = 1
Executive position	no = 0, yes = 1
Contact with customers	occasionally = 0, daily = 1

As a first step a saturated recursive path model was estimated for both “willingness to report to work” questions including the following paths:

- All exogenous variables - > perceived danger
- All exogenous variables - > competence
- All exogenous variables + perceived danger, competence − > perceived risk
- All exogenous variables + competence - > efficacy
- All exogenous variables + competence - > importance
- All exogenous variables + importance + efficacy + competence - > duty
- All exogenous variables + danger + perceived risk + competence + efficacy + importance - > willingness to report to work

In order to estimate indirect effects the total effects were deconstructed as follows:

- Competence - > efficacy - > perceived risk
- Competence - > importance - > duty
- Competence - > efficacy - > duty
- Danger - > perceived risk - > willingness to report to work
- Competence - > importance - > willingness to report to work
- Competence - > efficacy - > willingness to report to work
- Competence - > efficacy - > perceived risk - > willingness to report to work
- Competence - > importance - > duty - > willingness to report to work
- Competence - > efficacy - > duty - > willingness to report to work
- Age - > perceived risk - > willingness to report to work
- Age - > importance - > willingness to report to work
- Age - > duty - > willingness to report to work
- Age - > importance - > duty - > willingness to report to work

In order to find a parsimonious structure the model was re-estimated in a second step by removing all non-significant paths.

For assessing the fit of our models, we used the *χ*2 test, the Tucker Lewis index (TLI), the comparative fit index (CFI), the root mean square error of approximation (RMSEA), and the standardized root mean square residual (SRMR).

The TLI is defined as ((χ 2)/(df (null model))- *χ*2/(df (proposed model)))/(*χ*2(null model)) and should have a value > 0.95. The CFI is defined as (χ 2-df (null model)-χ 2-df (proposed model)) / (χ 2-df (null model)) and should also have a value > 0.95. Where the null model is a model where all structural (regression) paths between the model variables are assumed to be zero and the proposed model is the model to be tested. The RMSEA is defined as √((χ 2-df)/√(df-1)) where a value of 0.01 indicates an excellent fit, a value of 0.05 indicates a good fit and a value of 0.08 indicates a mediocre fit. The SRMR is the standardized difference between the empirically observed correlations between the model variables and the correlations predicted on the basis if the specified model and should have a value < 0.08.

For comparing the fit between the different models we used the Akaike Information Criterion (AIC) which is defined as *χ*2+ k(k-1)-2df, were k is the number of variables in the model, and the Bayesian Information Criterion (BIC) which is defined as *χ*2+ ln(N)[k(k-1)/2-df], where k is the number of variables in the model and ln(N) is the natural logarithm of the number of cases [31, 32].

Cases with missing values were excluded from the path analyses. Path models were computed with MPLUS 5.21, and all other statistical analyses were performed with STATA 13.

## Results

A total of 1.566 employees answered the survey. The overall response rate was 26 % of the total staff members. However, 44 (2.8 %) participants were excluded from further analyses because of missing demographic data. The characteristics of the remaining sample are presented in Table 2. On average the respondents were 45 years old (sd = 9.6 years) and 763 (50.1 %) were male. 1.035 (68 %) of the participating employees had a high school degree or above and 595 (39.45 %) had an executive position. 595 (39.33 %) had a civil servant status. Of those participants with a civil servant status 299 (50.5 %) had also an executive position. 673 (44.33 %) of the respondents stated that they had daily contact with citizens as part of their work.

**Table 2:** Study sample

n	1,566
Socio-demographic characteristics	
Age mean (sd)	45 (9.6)
Male gender n (%)	763 (50.1)
Higher education n (%)	1,035 (68.2)
Job features	
Executive position n (%)	595 (39.8)
Civil servant status n (%)	589 (39.6)
Daily contact with citizens n (%)	673 (44.3)

### Psychometric properties of the summary scales

As indicated by the Spearman-Brown reliability coefficients (Table 3) all EPPM summary scales have at least a sufficient internal consistency.

**Table 3:** Spearman-Brown reliability of the EPPM summary scales

EPPM Dimension	Number of items	Spearman-Brown reliability
Perceived danger of pandemic	2	0.70
Perceived personal risk	3	0.86
Role importance	3	0.80
Role competence	4	0.86
Self-efficacy	4	0.75
Sense of duty	2	0.61

### Willingness to report to work

1358 participants answered the questions about their willingness to report to work if required and 1.393 answered the question about their willingness to report to work if asked but not required. As indicated by Table 4 about 11 % of the municipal administration workers stated that they would not, or would rather not, report to work during an IP, even if they were required by their department. If they were only asked, but not required to report to work, the percentage of those who indicated that they would not report to work increased to 20 %.

**Table 4:** Willingness to report to work

	If I were required by my agency to report to work in an influenza pandemic, I would report	If I were asked, but not required by my agency to report to work in an influenza pandemic, I would report
	*n* (%)	*n* (%)
Not true at all	48 (3.53)	93 (6.68)
Rather does not agree	100 (7.36)	185 (13.28)
Partly agrees	163 (12,00)	276 (19.81)
Mainly true	288 (21.21)	364 (26.13)
Agrees completely	759 (55.89)	475 (34.10)
Missing values	208 (13.28)	173 (11.50)
Total	1,566 (100)	1,566 (100)

### Path model 1: Willingness to report to work if required by the department

**Fig. 1:**
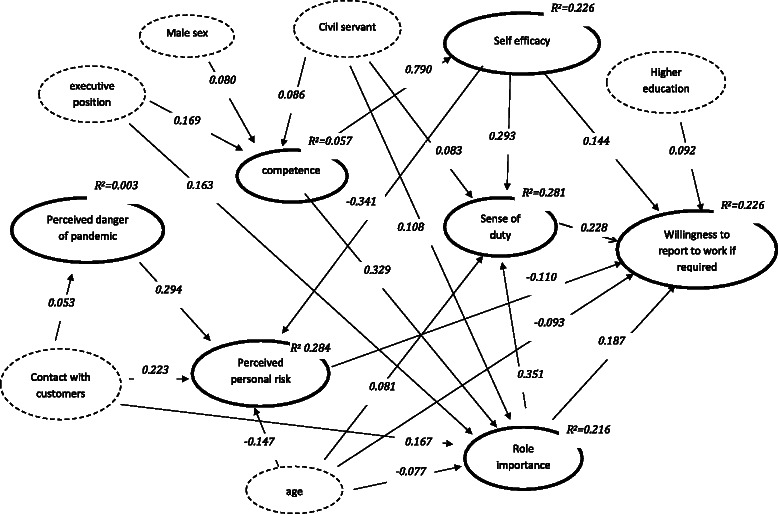
Path model for the willingness to report to work if required (standardised path coefficients for significant direct effects, p <= 0.05)

Figure 1 shows the standardised path coefficients of the restricted path model for the willingness to report to work if required by the agency.

### Factors affecting the perceived danger of pandemic

As indicated by the standardised path coefficient (ß = 0.053; 95 % CI = −0.104 to −0.002; p ≤ 0.050) staff members with daily customer contact assessed the danger of an IP higher than those who had only occasional contact with customers. The R^2^ reveals that 0.3 % of the variance in the assessment of danger was explained by the model.

### Factors affecting perceived personal risk

Staff members with daily customer contact assessed their own risk of becoming infected or carrying the infection into the family higher than those with a low frequency of contact (ß = 0.223; 95 % CI = 0.181 to 0.264; p ≤ 0.001). In addition, the perception of risk increased with the assessment of the danger that a pandemic will break out (ß = 0.294, 95 % CI = 0.252 to 0.336; p ≤ 0.001), but decreased with the estimation of self-efficacy (ß = −0.341, 95 % CI = −0.382 to −0.300; p ≤ 0.001) and with the participants’ age (ß = −0.147; 95 % CI = −0.190 to −0.104; p ≤ 0.001). Decomposition of total effects (not shown in Fig. 1) indicated a significant negative indirect effect from competence over efficacy to perceived risk (ß = −0.261; 95 % CI = −0.330 to −0.192; p ≤ 0.001). As indicated by the R^2^ the model explained 28.4 % of the variance in the perception of risk.

### Factors affecting perceived role competences

Standardised path coefficients revealed that participants in an executive position (ß = 0.169; 95 % CI = 0.119 to 0.218; p ≤ 0.001) and participants with a civil servant status (ß = 0.086; 95 % CI = 0.038 to 0.135; p ≤ 0.001) assessed their competence to fulfil their role requirements during a pandemic higher than those in lower positions or those without a civil servant status. Male participants assessed their competence higher than their female counterparts (ß = 0.080; 95 % CI = 0.031 to 0.130, p ≤ 0.001). As indicated by the R^2^, 5.7 % of the variance in the assessment of role competence was explained by the model.

### Factors affecting self-efficacy expectations

As indicated by the standardised path coefficient (ß = 0.790; 95 % CI = 0.609 to 0.971; p ≤ 0.001) self-efficacy expectations regarding the capability of performing effectively in a pandemic situation were only affected by the assessment of role competence, which explained 22.6 % of the variance.

### Factors affecting perceived role importance

The assessment of the importance of participants’ professional role increased with the estimation of their role competence (ß = 0.329; 95 % CI = 0.285 to 0.373; p ≤ 0.001). Staff members with daily customer contacts assessed their role during a pandemic as more important than those with occasional contact (ß = 0.167; 95 % CI = 0.122 to 0.212; p ≤ 0.001). Participants in executive positions (ß = 0.163; 95 % CI = 0.116 to 0.211; p ≤ 0.001) and those with a civil servant status (ß = 0.108; 95 % CI = 0.062 to 0.154, p ≤ 0.001) assessed their role importance higher than those in lower positions and those with an employee status. Older staff members assessed their professional role as less important than their younger colleagues (ß = −0.077; 95 % CI = −0.124 to −0.031, p ≤ 0.001). 21.6 % of the variance in the assessment of role importance was explained by the model.

### Factors affecting the sense of duty

With increasing self-efficacy (ß = 0.293; 95 % CI = 0.249 to 0.337; p ≤ 0.001) and an increasing estimation of their role importance (ß = 0.351; 95 % = CI 0.307 to 0.394, p ≤ 0.001), staff members also felt more responsible to fulfil their professional tasks during a pandemic. Participants with a civil servant status (ß = 0.083; 95 % = CI 0.039 to 0.127; p ≤ 0.001) and those at a higher age (ß = 0.081; 95 % CI = 0.037 to 0.125; p ≤ 0.001) felt more responsible to fulfil their tasks during a pandemic than ordinary employees and younger persons. Decomposition of total effects (not shown in Fig. 1) revealed indirect positive effects from competence over role importance (ß = 0.115; 95 % CI = 0.094 to 0.137; p ≤ 0.001) and from competence over efficacy (ß = 0.231; 95 % CI = 0.168 to 0.295; p ≤ 0.001) to sense of duty.

The model explained 28.1 % of the variance in the sense of duty.

### Factors affecting the willingness to report to work

Staff members’ willingness to report to work if required increased with increasing self-efficacy (ß = 0.144; 95 % CI = 0.087 to 0.201; p ≤ 0.001), sense of duty (ß = 0.228; 95 % CI = 0.172 to 0.283; p ≤ 0.001), and with increasing assessment of role importance (ß = 0.187; 95 % CI = 0.133 to 0.240; p ≤ 0.001). However, it decreased with an increasing perception of the risk to become infected or transmit the infection to a family member (ß = −0.110; 95 % CI = −0.162 to −0.059; p ≤ 0.001). While willingness to report to work increased with the level of education (ß = 0.092; 95 % CI = 0.044 to 0.193; p ≤ 0.001), it decreased with increasing age (ß = −0.093; 95 % CI = −0.141 to −0.045; p ≤ 0.001). Decomposition of total effects (not shown in Fig. 1) indicated negative indirect effects of perceived danger over perceived risk (ß = −0.032; 95 % CI = −0.017 to −0.048; p ≤ 0.001). Positive indirect effects of competence on willingness to report to work were identified over importance (ß = 0.061; 95 % = CI 0.042 to 0.081; p ≤ 0.001), efficacy (ß = 0.114; 95 % CI = 0.062 to 0.166; p ≤ 0.001), efficacy and perceived risk (ß = 0.030; 95 % CI = 0.014 to 0.046; p ≤ 0.001), role importance and sense of duty (ß = 0.026; 95 % CI = 0.018 to 0.034; p ≤ 0.001), and efficacy and sense of duty (ß = 0.053; 95 % CI = 0.033 to 0.072; p ≤ 0.001). Significant indirect effects on willingness to report to work were identified from age over perceived risk (ß = 0.016; 95 % CI = 0.007 to 0.025; p ≤ 0.001), role importance (ß = −0.014; 95 % CI = −0.024 to −0.005; p = 0.003), sense of duty (ß = 0.018; 95 % CI = 0.007 to 0.029; p ≤ 0.001), and over importance and sense of duty (ß = −0.006; 95 % CI = −0.010 to −0.002; p ≤ 0.010). Minor significant indirect effects on willingness to report to work were also obtained from gender over competence and role importance (ß = 0.005; 95 % CI = 0.002 to 0.008; p ≤ 0.010), competence and efficacy (ß = 0.009; 95 % CI 0.002 to 0.016; p ≤ 0.010), competence, efficacy, and perceived risk (ß = 0.002; 95 % CI 0.000 to 0.004, p ≤ 0.050), competence, role importance, and sense of duty (ß = 0.002; 95 % CI 0.001 to 0.004; p ≤ 0.010), and competence, efficacy and sense of duty (ß = 0.004; 95 % CI 0.001 to 0.007, p ≤ 0.010).

In total the model explained 22.6 % of the variance in participants’ willingness to report to work if required by the employing department.

### Path model 2: Willingness to report to work if requested but not required by the department

**Fig. 2:**
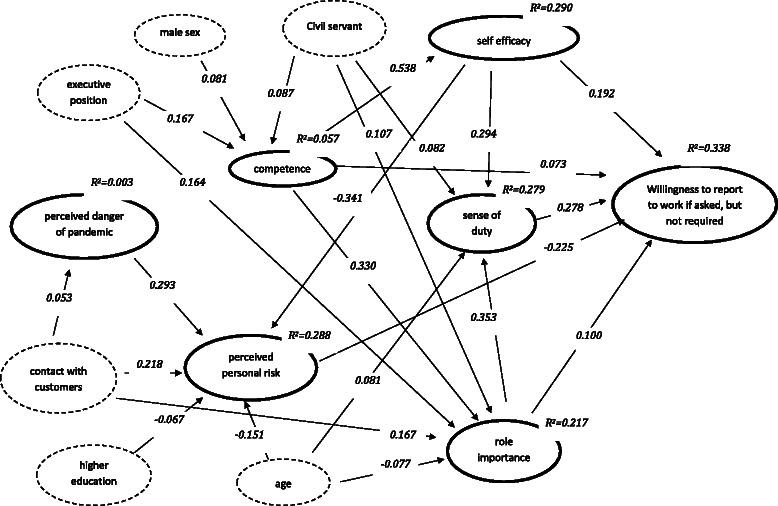
Path model for the willingness to report to work if asked, but not required (standardised path coefficients for significant direct effects, p <= 0.05)

With few exceptions the path coefficients and the R^2^ values of model 2 (Fig. 2) were largely identical to those of path model 1. As one major difference the effect of perceived role competence on perceived self efficacy was smaller in model 2 (ß = 0.538; 95 % CI = 0.503 to 0.573; p ≤ 0.001). Furthermore, in contrast to the willingness to report to work if required by the department, the willingness to report to work if only asked but not required was not affected by the participants’ educational level and age. Instead the willingness to report to work voluntarily was found to be more strongly negatively related to the perception of the risk to get infected or to pass on the infection to a family member (ß = −0.225; 95 % CI = −0.271 to −0.179 p ≤ 0.001). While the positive effect of the assessment of the importance of the professional role (ß = 0.100; 95 % CI = 0.049 to 0.151; p ≤ 0.001) was found to be lower than in model 1, the positive effects of competence (ß = 0.073; 95 % CI 0.019 to 0.126; p ≤ 0.010), sense of duty (ß = 0.278; 95 % CI = 0.228 to 0.328; p ≤ 0.001), and self-efficacy (ß = 0.192; 95 % CI = 0.134 to 0.250; p ≤ 0.001) were stronger.

Decomposition of total effects (not shown in Fig. 2) revealed an indirect negative effect from perceived danger to willingness to report to work over perceived risk (ß = −0.066; 95 % CI = −0.083 to −0.049; p ≤ 0.001). From competence indirect effects on willingness to report to work were identified over role importance (ß = 0.033; 95 % CI = 0.016 to 0.051; p ≤ 0.001), efficacy (ß = 0.103; 95 % CI = 0.071 to 0.136; p ≤ 0.001), efficacy and perceived risk (ß = 0.041; 95 % CI = 0.031 to 0.052; p ≤ 0.001), role importance and sense of duty (ß = 0.032; 95 % CI = 0.024 to 0.041 p ≤ 0.001), and efficacy and sense of duty (ß = 0.044; 95 % CI 0.033 to 0.055 p ≤ 0.001). Indirect effects of age were identified over perceived risk (ß = 0.034; 95 % CI = 0.022 to 0.046; p ≤ 0.001), role importance (ß = −0.008; 95 % CI = −0.014 to −0.002; p ≤ 0.010), sense of duty (ß = 0.023; 95 % CI = 0.010 to 0.035; p ≤ 0.001), and role importance and sense of duty (ß = −0.008; 95 % CI = −0.012 to −0.003; p ≤ 0.010). Significant indirect effects of gender were found over role competence (ß = 0.006; 95 % CI = 0.000 to 0.012; p ≤ 0.050), role competence and role importance (ß = 0.003; 95 % CI = 0.000 to 0.005; p ≤ 0.050), role competence and efficacy (ß = 0.008; 95 % CI = 0.002 to 0.014; p ≤ 0.010), role competence, efficacy and perceived risk (ß = 0.003; 95 % CI = 0.001 to 0.006; p ≤ 0.010), role competence, role importance, and sense of duty (ß = 0.003; 95 % CI = 0.001 to 0.004; p ≤ 0.010), and role competence, efficacy, and sense of duty (ß = 0.004; 95 % CI = 0.001 to 0.006; p ≤ 0.010).

The R^2^ value indicated that with 33.8 % a larger proportion of the variance in the willingness to report to work voluntarily was explained by the model than in the variance of the willingness to report to work if required by the department.

### Overall model fit

The parameters for the goodness of fit of the path models are presented in Table 5. The results of the Chi^2^ tests indicated a significant deviation of the empirical covariance structure from the structure hypothezised by the models. However, due to the large sample size the Chi^2^ is not a good indicator of the model fit and the global fit parameters CFI and TLI as well as the RMSEA and the SRMR are within the range of an acceptable fit for both models. The comparison of the information criteria AIC and BIC indicated that there is no difference in the fit of both models.

**Table 5:** Fit indices of the path models

	Willingness to report to work if required	Willingness to report to work if asked but not required
Chi^2^	173.17	174.95
Degrees of freedom	38	39
P value	0.000	0.000
CFI	0.947	0.951
TLI	0.912	0.921
AIC	48083.01	48244.76
BIC	48291.86	48448.25
RMSEA	0.048	0.047
SRMR	0.029	0.029

## Discussion

To our knowledge this is the first study that investigated the factors that affect the non-medical municipal employees’ willingness to report to work during the event of an IP. Similar to studies on medical service workers [12, 13, 15–17, 25, 27, 28] about 11 % of the municipal employees expressed that they would not or rather not report to work if they are required by their department during an IP, while about 20 % would not report to work if they were only asked but were not required by their department. However, our results also revealed that only 56 % of the employees stated that they would definitively report to work if required and 34 % that they would definitively report if asked but not required. Considering the fact that the clinical attack rate of the working population in the case of an IP is estimated between 25 % and 50 % [33, 34], the prophylactic absenteeism would cause another 10-20 % of the employees to stay at home, and this could cause a serious labour shortage in municipal services.

Findings of our study indicate that the extended parallel process model (EEPM) provides a suitable theoretical basis for the investigation of factors affecting the willingness of municipal employees to report to work during an IP. As our most important result we found that the municipal employees’ assessment of their professional competence and their self-efficacy expectations (competence – efficacy dimension) as well as their assessment of the importance of their professional role and their sense of duty toward the citizens (importance – sense of duty dimension) were more important predictors of their willingness to report to work than their assessment of the probability and dangerousness of the pandemic and their perceived risk to become infected or pass on the illness to a family member (threat – susceptibility dimension). The identified relationships are in accordance with the propositions of the EPPM that perceived competences and self-efficacy expectations are crucial cognitions in the process of adaptive reactions to external threats [16, 21].

The differences between the models indicated that in the more voluntary scenario the perceived threat and perceived susceptibility had stronger negative effects while perceived role competence, perceived self-efficacy and the sense of duty had stronger positive effects on the willingness to report to work if asked but not required than on the willingness to report if required. On the other hand, perceived role importance had a stronger effect on the willingness to report if required.

In total our results suggest that the willingness of municipal employees to report to work in the event of an IP is affected by the same variables as those identified for local public health workers [12, 13, 15], for hospital workers [17, 25], for emergency medical service workers [14] and for medical reserve corps volunteers [27].

However, due to the application of path analyses our study provides additional new insights into the relationships between the factors of the EPPM. So, the importance of role competence and self-efficacy expectations in the process of improving the willingness to report to work becomes clearer by the fact that beyond its direct effect role competence has a strong indirect positive effect on willingness to report to work by increasing self-efficacy expectations. Moreover, self-efficacy expectations improve the willingness to report to work not only directly but also indirectly by decreasing feelings of susceptibility and by increasing the sense of duty. Willingness to report to work was also indirectly affected by the features of the professional position. Employees in executive positions and those with a civil servant status assessed their role competence and their role importance higher than other employees. In addition, those who had a civil servant status expressed a higher sense of duty than those with a normal employee status. These results are in agreement with the expectation that people in superior positions feel more competent and more important than those who are lower in the hierarchy. In addition, the higher sense of duty in participants with a civil servant status is in accordance with the requirements of mandatory loyalty and compulsory service, which are related to the civil servant status according to the German Civil Servant Law [35]. Two contradictory indirect effects were identified for the frequency of customer contacts. A higher frequency of customer contact was found to be related to a higher assessment of danger and perceived personal risk, which is causing an indirect negative effect on the willingness to report to work, while increased customer contact was also found to be related to an increased perceived role importance causing an indirect positive effect on willingness to report to work.

With few exceptions, socio-demographic characteristics were related to willingness to report to work indirectly via perceived risk and the assessment of role competences and professional role importance. We found that male participants independently of their job position and their status as civil servants assessed their role competences higher than their female counterparts. These findings correspond to studies on gender related bias in the self-assessment of task competence indicating that due to gender role stereotypes women estimate their instrumental task competence lower than man, even if they have the same qualifications or professional positions [36, 37]. Two contradictory effects were also obtained for age. On the one hand, increasing age was directly related to decreased willingness to report to work if required and in addition willingness to report to work was in both cases indirectly negatively affected by age via negative effects on perceived role importance. On the other hand, age was indirectly positively related in both cases to willingness to report to work via a negative effect on perceived risk and a positive effect on sense of duty. While the positive effect of age on sense of duty can be interpreted as a generation effect related to the decreasing societal role of virtues such as conscientiousness in modern societies [38, 39], the negative effect of age on perceived role importance could indicate that with increasing age employees feel less respected with regard to their working task performance. More difficult to explain are the negative effects of age on perceived risk and the fact that age is directly negatively related to the willingness to report to work if required but not to the willingness to report to work if only asked. Since the perceived risk and the dangerousness of an influenza infection increases with age, the negative age effect on perceived risk is not plausible. However, studies from several countries revealed that the perceived risk of an infection during an IP is underestimated in the general population and particularly in elderly people [40].

## Conclusions

According to the findings from studies on health services workers, it can be concluded from our study results that improving the willingness of municipal employees to report to work during a pandemic requires measures to inform staff members clearly about their professional role in managing the situation but also to promote their professional competences and their expectations with regard to fulfilling their tasks, even under the circumstances of a pandemic. This is particularly important for employees with frequent customer contact. In order to increase the willingness of these employees to report to work during an IP, measures for infection control in the customer areas of public buildings and for training the employees working in these areas to protect themselves from infection should be taken. With regard to age and gender it is necessary to address gender and age role stereotypes in measures for the improvement of the willingness to report to work. More efforts should be made to strengthen the trust of female staff in their professional skills and to prevent older employees from the impression that they are not needed in an emergency situation only due to their increased age.

Since the respondents’ assessment of the probability and the severity of an IP did not affect their willingness to report to work, there is no need for intensifying the threat of an IP. However, in order to reduce contagion rates and to increase the motivation for vaccination it seems necessary to emphasize the high infection risk and the high perceived risk of severe consequences, particularly for elderly employees.

### Limitations

The study was limited to the scenario of an IP. Studies on the willingness of health services staff to report have revealed significant differences between willingness to report during an IP and other scenarios such as weather related disaster, bioterrorism, and dirty bomb. Our study provides no information about such differences.

The use of an online survey via the intranet of the city administration limited the study participation mainly to employees with computer access during their working time. Due to protection of data privacy it was not possible to include questions concerning the different departments and their special duties and responsibilities. Therefore we had no possibility to check the representativeness of the sample with regard to the spectrum of municipal functions.

An important limitation of all studies in this field is the fact that willingness to report to work in such a scenario can only be assessed hypothetically.

## Appendix 1

**Table 6:** Items and Dimensions of the Administrative Staff Willingness to Report to Work Questionnaire

Dimension	Item
Willingness to report to work if required by the department	If I were required by my agency to report to work in an influenza pandemic, I would report.
Willingness to report to work if asked but not required by the department	If I were asked, but not required by my agency to report to work in an influenza pandemic, I would report.
Perceived danger of pandemic	If an influenza pandemic in this area occurs, there will be serious consequences for public health.
	In the next years there will be a high probability of an influenza pandemic in this area.
Perceived personal risk	My personal risk of infection in the context of an influenza pandemic is high.
	I would be afraid to get infected at work during an influenza pandemic.
	During an influenza pandemic I would be worried to bring the illness home with me and transmit it to my family.
Role importance	In case of an influenza pandemic my employer will ask me to report to work.
	My role in the city administration’s overall response to a pandemic flu emergency is important.
	With my work I could make an important contribution to the maintenance of the public order during a pandemic situation.
Role Competence	I know about possible impacts of an influenza pandemic for public health.
	I know about my specific occupational responsibilities during an influenza pandemic.
	I have the skills to perform my occupational responsibilities during an influenza pandemic.
	I am psychologically prepared to perform my occupational responsibilities during an influenza pandemic.
Self-efficacy	I am confident that I could get safely to work during a pandemic emergency.
	I would be able to perform my duties successfully in the event of an influenza pandemic.
	During an influenza pandemic I would also take on responsibilities outside of my normal job range.
	I would be able to address the questions of a member of the public during an influenza pandemic.
Sense of duty	As long as I’m not sick myself, it is my duty to report to work during an influenza pandemic.
	As an employee of the public administration, I have a special responsibility for the citizens during an influenza pandemic.
