# Erratum to: ‘The analysis of factors affecting municipal employees’ willingness to report to work during an influenza pandemic by means of the extended parallel process model (EPPM)’

**DOI:** 10.1186/s12889-016-2869-4

**Published:** 2016-07-20

**Authors:** Carolin von Gottberg, Silvia Krumm, Franz Porzsolt, Reinhold Kilian

**Affiliations:** Department of Psychiatry and Psychotherapy II, Section of Health Economics and Mental Health Services Research, Ulm University, Ludwig-Heilmeyer-Str. 2, D-89315 Günzburg, Germany; Department of General and Visceral Surgery, Working Group of Health Services Research, Ulm University, Albert-Einstein-Allee 23, D-89070 Ulm, Germany

## Erratum

Unfortunately, the original version of this article [1] contained missing graphics of the path models. This has now been included correctly below.

**Fig. 1:**
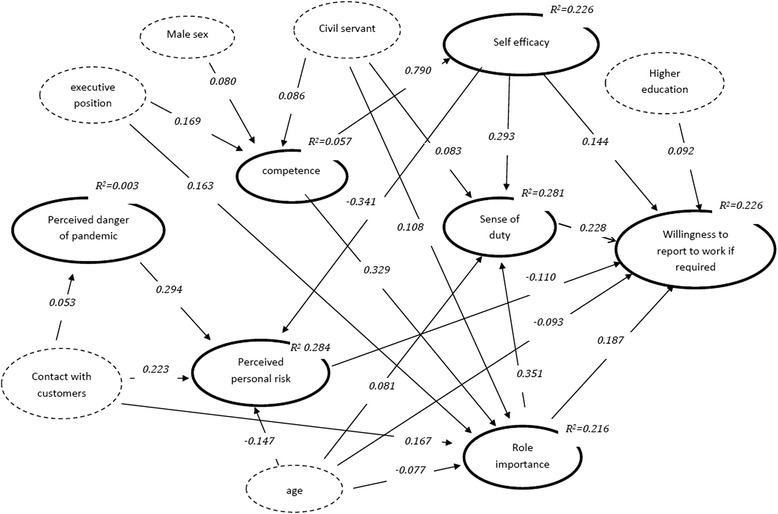
Path model for the willingness to report to work if required (standardised path coefficients for significant direct effects, *p* < =0.05)

**Fig. 2:**
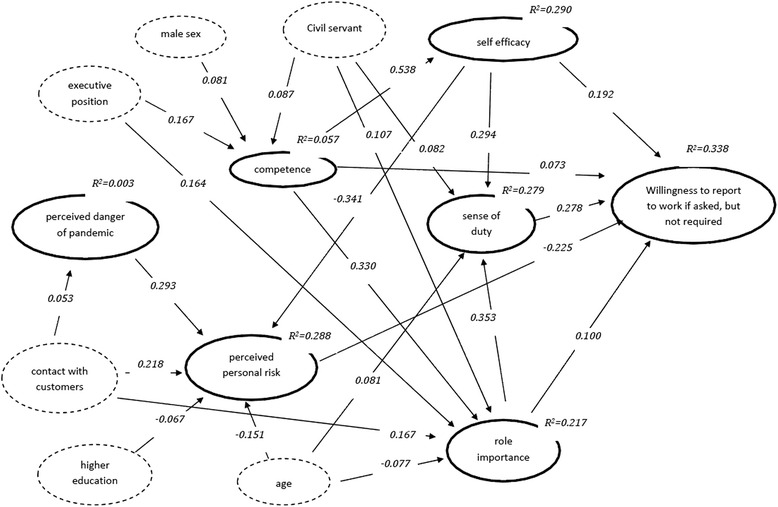
Path model for the willingness to report to work if asked, but not required (standardised path coefficients for significant direct effects, *p* < =0.05)
